# Differences in Lactation Performance, Rumen Microbiome, and Metabolome between Montbéliarde × Holstein and Holstein Cows under Heat Stress

**DOI:** 10.3390/microorganisms12081729

**Published:** 2024-08-22

**Authors:** Hantong Weng, Hanfang Zeng, Haihui Wang, Haomiao Chang, Yunfei Zhai, Shujie Li, Zhaoyu Han

**Affiliations:** College of Animal Science and Technology, Nanjing Agricultural University, Nanjing 210095, China

**Keywords:** dairy cows, heat stress, crossbreeding, bacteria, metabolites

## Abstract

Heat stress negatively affects lactation performance and rumen microbiota of dairy cows, with different breeds showing varying levels of heat tolerance. This study aimed to compare the lactation performance of Montbéliarde × Holstein (MH, *n* = 13) and Holstein (H, *n* = 13) cows under heat stress, and 16S rRNA sequencing and liquid chromatography–mass spectrometry (LC-MS) were used to determine the rumen microbiome and metabolome in experimental cows. The results indicated that during heat stress, milk yield (*p* = 0.101), milk fat yield, milk protein yield, milk protein, and milk lactose (*p* < 0.05) in Montbéliarde × Holstein cows were higher than those in Holstein cows, whereas milk yield variation and somatic cell counts (*p* < 0.05) were lower than those in Holstein cows. The sequencing results indicated that the rumen of Montbéliarde × Holstein cows was significantly enriched with beneficial bacteria, such as Rikenellaceae, *Allobaculum*, and *YRC22* (*p* < 0.05). In addition, correlations were observed between specific ruminal bacteria and lactation performance. Ruminal metabolites related to antioxidant and anti-inflammatory properties were significantly higher (*p* < 0.05) in Montbéliarde × Holstein cows than in Holstein cows. Overall, Montbéliarde × Holstein cows showed higher production efficiency under heat stress, which may be related to the different rumen mechanisms of crossbred and Holstein cows in adapting to heat stress.

## 1. Introduction

With the continuous increase in greenhouse gas (GHG) emissions in recent years, global warming has severely restricted livestock production and development, particularly dairy production. Heat stress can decrease the feed intake of dairy cows [1], affect lactation performance [2], induce oxidative stress [3], and alter ruminal microbial composition and metabolism [4], leading to a decline in the health status of dairy cows. Holstein cows, the main global dairy cow breed, are characterized by large body size, small surface area for heat dissipation per unit weight, and underdeveloped sweat gland functions. Furthermore, there is a significant increase in the heat generated by metabolism and lactation with the enhancement of production performance [5]. Therefore, inadequate heat dissipation and elevated body heat production aggravate the effects of heat stress in Holstein cows.

Crossbreeding improves the economic benefits of dairy farming, mainly manifested in milk production performance, reproductive performance, and disease resistance [6]. Crossbreeding can be used as an effective strategy for enhancing heat tolerance in dairy cows [7]. For example, the offspring of Holstein cows crossed with Gir [8], dairy buffaloes [9], Jersey [10], and Brown Swiss [11] have excellent heat resistance. As one of the main dairy breeds in France, Montbéliarde cows exhibit excellent milk production and growth performance, high adaptability and fertility, and a long service life [12]. Currently, Montbéliarde cows are crossbred with Holstein cows to improve their performance and profitability, resulting in crossbred cows with higher milk fat and protein contents and a reduced risk of mastitis [13]. However, the existing research has rarely addressed the differences in lactation performance and rumen microbiota between Montbéliarde × Holstein and Holstein cows under heat stress.

The rumen is an important organ for digestion and absorption in ruminants and contains rich microbial communities. These microorganisms can degrade plant fibers and produce volatile fatty acids (VFAs), which can provide 60–70% of the metabolizable energy of the host, thus affecting the productivity of animals and the quality of livestock products [14]. The rumen microbiota of cows is highly sensitive to temperature changes. Heat stress induces changes in the rumen microbial community composition and diversity, thereby affecting microbial metabolism and function [15]. Previous studies using 16S rRNA have found that heat stress alters the abundance of specific bacteria in the rumen of Holstein dairy cows [16]. Rumen metabolites are associated with microorganisms, and metabolomics better explains the phenotypic changes than genomics or proteomics. Researchers used liquid chromatography–mass spectrometry(LC-MS) to determine blood and rumen metabolome changes in heat-stressed cows at different growth stages and found that heat stress resulted in significant enrichment of fatty acyl metabolites [17]. Therefore, 16S rRNA sequencing and metabolomics could further reveal the effects of heat stress on ruminal microorganisms in dairy cows.

Our previous study compared the differences in milk performance throughout the lactation cycle between Montbéliarde × Holstein and Holstein cows, as well as the differences in rumen microbiota and metabolome under non-heat-stress conditions [18]. In this study, we compared physiological parameters and lactation performance under heat stress and analyzed rumen microbiota and metabolism in Montbéliarde × Holstein and Holstein cows. The aim of this study was to investigate the relationship between rumen microorganisms and the lactation performance of heat-stressed dairy cows.

## 2. Materials and Methods

All experimental procedures were carried out following the Institutional Animal Care and Use Committee of Nanjing Agricultural University, China (SYXK 2011-0036).

### 2.1. Animals

The experiment was conducted at the Xuzhou Weigang Animal Husbandry of Jiangsu Province, Xuzhou, China. Based on the principle of similar lactation time, parity, and milk yield, 13 Montbéliarde × Holstein cows (MH, DIM (days in milk) = 82.92 ± 6.97 d, parity = 1.62 ± 0.21) were selected as the experimental group and 13 Holstein cows (H, DIM = 85.15 ± 6.45 d, parity = 1.62 ± 0.14) as the control group. A month preceding the experiment, the milk yield was 43.05 ± 2.04 kg/d for Montbéliarde × Holstein cows and 43.12 ± 1.79 kg/d for Holstein cows. All cows were kept under the same conditions. The cowsheds were equipped with fans and sprinkler systems. The cows were fed total mixed rations (TMR) of the same formula. They were fed and milked three times daily and had free access to water throughout the study, i.e., July and August.

### 2.2. Sample Collection

The temperature–humidity index (THI) was measured three times daily (at 0800 h, 1400 h, and 2000 h) during the test period and was calculated as follows [19]:

THI = (1.8 × Temperature + 32) − (0.55 − 0.55 × %Relative Humidity) × (1.8 × Temperature − 26)(1)

The rectal temperature and respiratory rate were measured every ten days at 1500 h for five measurements. An animal thermometer was used to measure the rectal temperature, and a stopwatch was used to count the number of chest undulations within a minute to measure respiratory rate. Milk yield and milk composition were measured every two weeks for four measurements. The DHI online detection system (Bentley NexGen FCM-FTS, United States) was used to determine milk fat (%), milk protein (%), milk lactose (%), milk total solids (%), milk urea nitrogen (mg/dL), and somatic cell counts (10^4^/mL). Yields of 4% fat-corrected milk (FCM) [20] and milk components were calculated using milk yield and component concentrations.

All samples were collected on 21 August. Seven cows were randomly selected from each group to collect rumen fluid after 2 h of morning feeding using a rumen oral catheter. The first 100 mL of rumen fluid was discarded to avoid saliva contamination. Subsequently, we collected 100 mL of rumen fluid and filtered it through four layers of sterile gauze. Rumen fluid was preserved in liquid nitrogen for subsequent determination of rumen microbiome and metabolome.

### 2.3. 16S rRNA Sequencing

Total genome DNA from the rumen fluid was extracted using cetyltriethylammonium bromide. The quality of DNA extraction was detected by 1.2% agarose gel electrophoresis. Primers 338F (5′-ACTCCTACGGGGAGGCAGCA-3′) and 806R (5′-GGACTACHVGGGTWTCTAAT-3′) were used to amplify the V3-V4 of the 16S rRNA region of 16S rRNA. The amplification parameters were set as follows: initial denaturation at 98 °C for 2 min, followed by 30 cycles of denaturation at 98 °C for 15 s, annealing at 55 °C for 30 s, and extension at 72 °C for 30 s. Finally, 72 °C for 5 min. Subsequent sequencing was handed over to PANOMIX (Suzhou, China).

Microbiome bioinformatics was performed with QIIME 2 2019.4 with slight modifications, according to the official tutorials (https://docs.qiime2.org/2019.4/tutorials/ (accessed on 29 March 2024)). Briefly, raw sequence data were demultiplexed using the demux plugin, followed by primers cutting with the cutadapt plugin. Sequences were then quality-filtered, denoised, merged, and chimera-removed using the DADA2 plugin [21]. Non-singleton amplicon sequence variants were aligned with mafft and used to construct a phylogeny with fasttree2 [22]. Alpha-diversity metrics and beta-diversity metrics were estimated using the diversity plugin. Principal coordinate analysis (PCoA) was performed to get principal coordinates and visualize from complex, multidimensional data, which were displayed by R software (Version 2.15.3).

### 2.4. LC-MS Analysis

The rumen fluid was thawed at 4 °C, vortexed for 1 min after thawing, and mixed evenly. An appropriate amount of sample was accurately transferred into a 2 mL centrifuge tube, then 400 µL methanol was added, and the tube was vortexed for 1 min. It was then centrifuged for 10 min at 12,000 rpm and 4° C, and then all the supernatant was transferred to a new 2 mL centrifuge tube and concentrated to dry it. Then, 150 µL of 2-chloro-l-phenylalanine (4 ppm) solution (prepared with 80% methanol–water) was added to redissolve the sample, and the supernatant was filtered using a 0.22 μm membrane and then transferred into the detection bottle for LC-MS detection [23].

The LC analysis was performed on a Vanquish UHPLC System (Thermo Fisher Scientific, Waltham, MA, USA). Chromatography was carried out with an ACQUITY UPLC ^®^ HSS T3 (2.1 × 100 mm, 1.8 µm) (Waters, Milford, MA, USA). The column was maintained at 40 °C. The flow rate and injection volume were set at 0.3 mL/min and 2 μL, respectively. For LC-ESI (+)-MS analysis, the mobile phases consisted of (B2) 0.1% formic acid in acetonitrile (*v*/*v*) and (A2) 0.1% formic acid in water (*v*/*v*). Separation was conducted under the following gradient: 0–1 min, 8% B2; 1–8 min, 8–98% B2; 8–10 min, 98% B2; 10–10.1 min, 98–8% B2; 10.1–12 min, 8% B2. For LC-ESI (−)-MS analysis, the analytes were carried out with (B3) acetonitrile and (A3) ammonium formate (5 mM). Separation was conducted under the following gradient: 0–1 min, 8% B3; 1–8 min, 8–98% B3; 8–10 min, 98% B3; 10–10.1 min, 98–8% B3; 10.1–12 min, 8% B3 [24].

Mass spectrometric detection of metabolites was performed on Orbitrap Exploris 120 (Thermo Fisher Scientific, USA) with an ESI ion source. Simultaneous MS1 and MS/MS (Full MS-ddMS2 mode, data-dependent MS/MS) acquisition was used. The parameters were as follows: sheath gas pressure, 40 arb, aux gas flow, 10 arb; spray voltage, 3.50 kV, and −2.50 kV for ESI(+) and ESI(−), respectively; capillary temperature, 325 °C, MS1 range, m/z 100–1000, MS1 resolving power, 60,000 FWHM; number of data-dependent scans per cycle, 4; MS/MS resolving power, 15,000 FWHM; normalized collision energy, 30%; dynamic exclusion time, automatic [25].

The raw data were first converted to mzXML format by MSConvert in the ProteoWizard software package (v3.0.8789) [26] then processed using R XCMS (v3.12.0) for feature detection [27], retention time correction, and alignment. Key parameter settings were as follows: ppm = 15, peakwidth = c (5, 30), mzdiff = 0.01, method = centWave. The batch effect was then eliminated by correcting the data based on QC samples. Metabolites with RSD > 30% in QC samples were filtered and then used for subsequent data analysis.

The R ropls (v1.22.0) package was used for partial least squares discrimination analysis (PLS-DA). Metabolites with variable importance in projection (VIP) value > 1.0 and *p*-value < 0.05 were considered differential metabolites. log2FC > 0 was used to represent upregulation of metabolites, and log2FC < 0 was used to represent downregulation of metabolites.

### 2.5. Statistical Analysis

The assumptions of normality and homogeneity of variance of the data were analyzed using the Shapiro–Wilk and Levene tests. The data on physiological parameters and lactation performance were analyzed using the Student’s *t*-test. The data on the rumen microbiome were analyzed using the Mann–Whitney U-test. Statistical analyses were performed using SPSS (version 26.0, SPSS Inc., Chicago, IL, USA), and graphs were generated using GraphPad Prism version 9.5.0 (GraphPad Software, San Diego, CA, USA). The results are presented as mean ± standard error or median (interquartile range), and a significance level of *p* < 0.05 was considered statistically significant. Additionally, Spearman’s correlation analysis was performed on rumen microbiota, lactation performance, and rumen fluid metabolites using the BioDeep platform (https://www.biodeep.cn (accessed on 30 May 2024)).

## 3. Results

### 3.1. THI and Physiological Parameters

**Figure 1:**
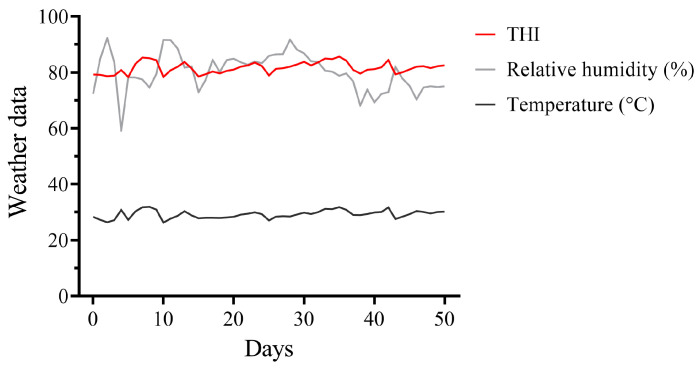
Temperature, relative humidity, and THI values (mean/d) of the cowsheds during the experimental period.

The THI is commonly used to assess the level of heat stress in dairy cows. When the THI is less than 72, cows exhibit no heat stress response; when the THI ranges from 72 to 79, cows experience mild heat stress; when the THI ranges from 80 to 89, cows experience moderate heat stress; and when the THI exceeds 90, cows experience severe heat stress [28]. During this experiment, the average temperature of the environment was 29.26 °C, the average relative humidity was 80.13%, and the average THI was 81.70 (Figure 1). The physiological parameters under heat stress are demonstrated in Table 1. Montbéliade × Holstein cows had significantly lower respiratory rates and rectal temperatures than Holstein cows (*p* < 0.05).

### 3.2. Lactation Performance

Table 2 shows the production performance of Montbéliade × Holstein and Holstein cows under heat stress. Montbéliade × Holstein and Holstein cows produced essentially the same amount of milk in one month preceding the experiment, whereas Montbéliade × Holstein cows had higher milk yield (*p* = 0.101) and 4% FCM (*p* < 0.05) than Holstein cows under heat stress. Furthermore, milk yield variation was significantly lower (*p* < 0.05) in Montbéliade × Holstein cows than in Holstein cows. In terms of milk composition, the milk fat yield, milk protein yield, milk protein rates, and milk lactose rates were higher (*p* < 0.05) in Montbéliade × Holstein cows than in Holstein cows, and milk somatic cell counts were significantly lower (*p* < 0.05) in Montbéliade × Holstein cows than in Holstein cows under heat stress.

### 3.3. Rumen Microbiome

**Figure 2:**
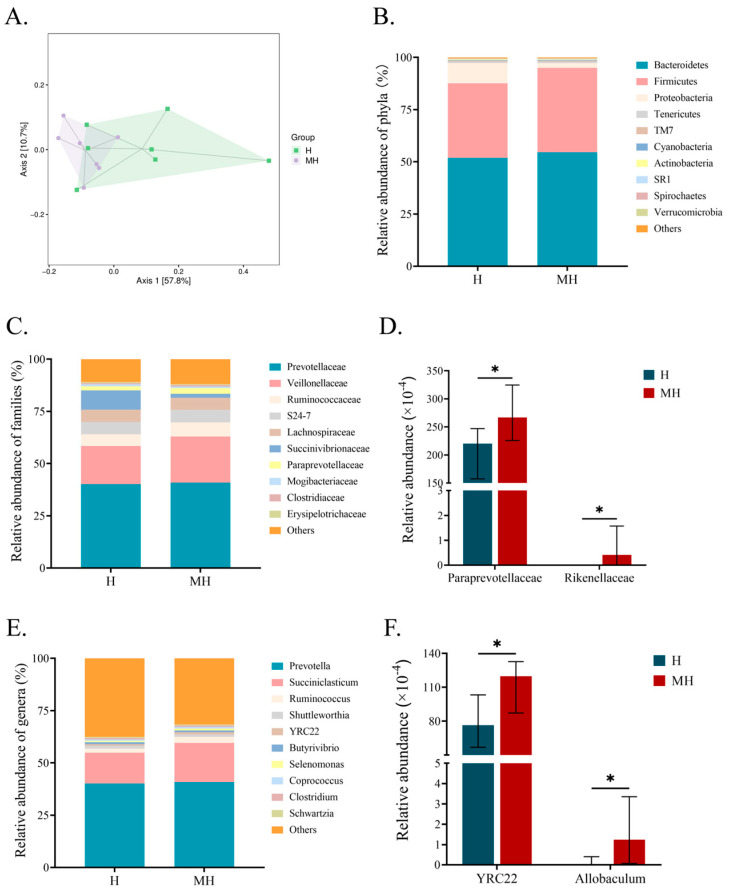
Results of 16S rRNA read sequence of the rumen bacteria between Montbéliade × Holstein and Holstein cows under heat stress. Principal coordinate analysis (PCoA) of rumen bacteria (**A**). Top 10 phyla (**B**), families (**C**), and genera (**E**). Significantly different relative abundances of bacterial families (**D**) and genera (**F**). Data are presented as median with 95% confidence interval (**D**,**F**). * *p* < 0.05.

In this study, we identified and analyzed bacteria in the rumens of Montbéliarde × Holstein and Holstein cows under heat stress using 16S rRNA sequencing. In terms of α-diversity, there was no significant difference in Goods_coverage, Simpson, Shannon, and Chao1 indices between Montbéliarde × Holstein and Holstein cows (Table 3). The PCoA plot showed the distribution of rumen bacteria in the two groups, and there was a tendency for the two groups to separate (Figure 2A).

The relative abundances of bacteria at the phylum, family, and genus levels are shown in Figure 2. Bacteroidetes, Firmicutes, and Proteobacteria were the dominant phyla in the rumen (relative abundance > 1%) (Figure 2B). The relative abundance of Proteobacteria in Montbéliarde × Holstein was higher (*p* = 0.119) than that in Holstein cows. At the family level, Prevotellaceae and Veillonellaceae were the dominant bacteria in the dairy cows (Figure 2C). A comparison of differences in relative abundance at the family level showed that Paraprevotellaceae and Rikenellaceae were significantly more abundant (*p* < 0.05) in Montbéliarde × Holstein cows than in Holstein cows (Figure 2D). The dominant genera in the rumen were *Prevotella*, *Succiniclasticum*, *Ruminococcus*, and *Shuttleworthia* (relative abundance > 1%) (Figure 2E). A comparison of the differences in relative abundance at the genus level showed that *Allobaculum* and *YRC22* were significantly more abundant (*p* < 0.05) in Montbéliarde × Holstein cows than in Holstein cows (Figure 2F).

### 3.4. Rumen Metabolome

**Figure 3:**
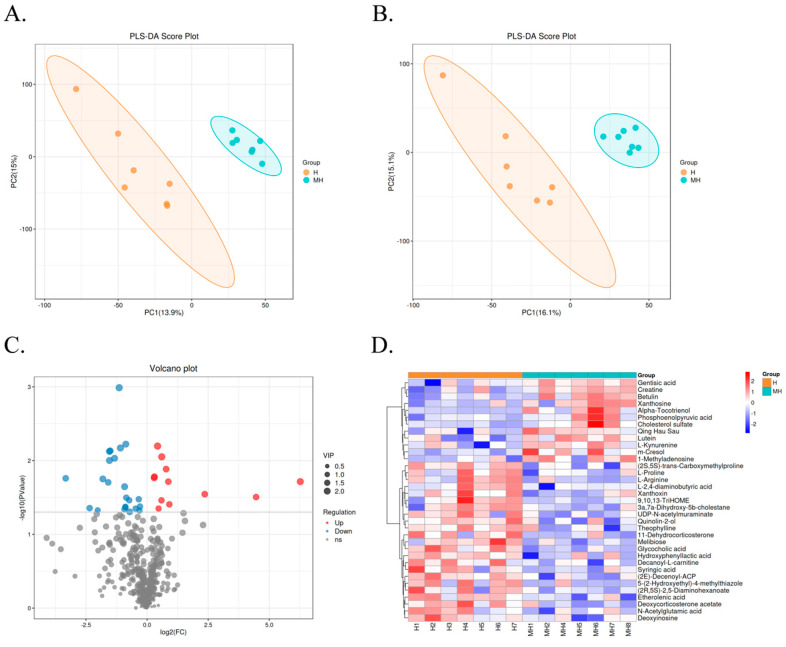
Differences in rumen fluid metabolite profiles between Montbéliarde × Holstein and Holstein cows under heat stress. Partial least squares discrimination analysis (PLS-DA) score plots of rumen metabolites of Montbéliade × Holstein and Holstein cows in both positive-ion (**A**) and negative-ion (**B**) modes. Volcano diagrams (**C**) and heat map (**D**) of differential metabolites.

In this study, LC-MS was used to identify and analyze the metabolites in the rumen fluid of Montbéliarde × Holstein and Holstein cows under heat stress. As shown in Figure 3A,B, PLS-DA revealed a clear separation of rumen fluid metabolites between the two groups. A total of 35 differential metabolites were identified (Figure 3D), of which the upregulated metabolites included m-cresol, creatine, L-kynurenine, Qing Hau Sau, 1-methyladenosine, alpha-tocotrienol, betulin, lutein, gentisic acid, phosphoenolpyruvic acid, xanthosine, and cholesterol sulfate. The downregulated metabolites included L-proline, L-2,4-diaminobutyric acid, (2E)-decenoyl-ACP, 5-(2-hydroxyethyl)-4-methylthiazole, (2R,5S)-2,5-diaminohexanoate, quinolin-2-ol, (2S,5S)-trans-carboxymethylproline, L-arginine, theophylline, xanthoxin, etherolenic acid, decanoyl-L-carnitine, 9,10,13-triHOME, 3a,7a-dihydroxy-5b-cholestane, hydroxyphenyllactic acid, N-acetylglutamic acid, syringic acid, deoxyinosine, 11-dehydrocorticosterone, melibiose, deoxycorticosterone acetate, glycocholic acid, and UDP-N-acetylmuraminate.

### 3.5. Correlation Analysis between Rumen Bacteria, Lactation Performance, and Metabolites

**Figure 4:**
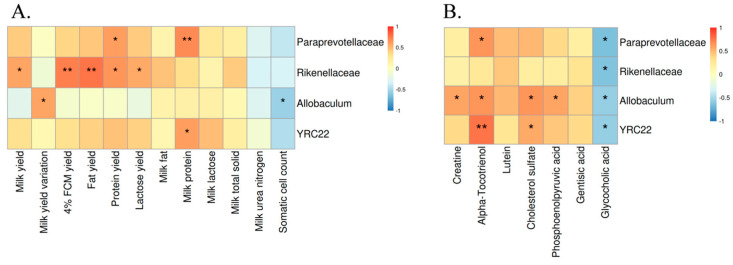
Spearman correlation analysis of rumen bacteria and lactation performance (**A**). Spearman correlation analysis of rumen bacteria and metabolites (**B**). * *p* < 0.05, ** *p* < 0.01.

We performed Spearman’s correlation analysis between differentially expressed bacteria and production performance. As shown in Figure 4A, Paraprevotellaceae was significantly positively correlated with protein yield and milk protein. Rikenellaceae was significantly positively correlated with milk yield, 4% FCM yield, fat yield, protein yield, and lactose yield. *Allobaculum* was significantly positively correlated with milk yield variation and significantly negatively correlated with somatic cell count. *YRC22* was significantly positively correlated with milk protein.

In addition, there were clear correlations between the differentially expressed bacteria and the differentially expressed metabolites of interest. As shown in Figure 4B, Paraprevotellaceae was significantly positively correlated with one metabolite and significantly negatively correlated with one metabolite. Rikenellaceae was significantly negatively correlated with one metabolite. *Allobaculum* was significantly positively correlated with four metabolites and significantly negatively correlated with one metabolite. *YRC22* was significantly positively correlated with two metabolites and significantly negatively correlated with one metabolite.

## 4. Discussion

### 4.1. Differences in Lactation Performance between Montbéliarde × Holstein and Holstein Cows under Heat Stress

During this experiment, the average THI inside the cowshed exceeded 80, indicating that the dairy cows experienced moderate heat stress. The exposure of dairy cows to prolonged high-temperature and high-humidity conditions can induce heat stress, leading to alterations in thermoregulation and elevation of the respiratory rate [29]. Based on our research findings, the rectal temperature and respiratory rate of Montbéliarde × Holstein cows was significantly lower than that of Holstein cows, indicating a more moderate response to extreme environmental temperature and humidity in Montbéliarde × Holstein cows.

Elevated temperatures can lead to a reduction in milk yield, milk fat, and milk protein in dairy cows while increasing somatic cell counts [30]. Lactating cows require a substantial quantity of feed to sustain milk production; however, diminished appetite resulting from heat stress frequently leads to inadequate fulfillment of energy requirements in lactating cows. In our study, we found that Montbéliarde × Holstein cows had higher milk yield and better milk quality under heat stress, such as milk fat, milk protein, and milk lactose. Additionally, the lower milk yield loss in Montbéliarde × Holstein cows meant that they were less affected by heat stress. Heat stress is one of the key triggers of mastitis in dairy cows [31]. The somatic cell count is a critical indicator for assessing the udder health of dairy cows. An increase in somatic cell counts during heat stress can further lead to a reduction in milk production [32]. We observed a lower somatic cell count in Montbéliarde × Holstein cows during heat stress, indicating superior disease resistance and immunity under such conditions.

### 4.2. Differences in Rumen Microbiome and Metabolome between Montbéliarde × Holstein and Holstein Cows under Heat Stress

Rumen microbiome and its metabolome play an important role in individualized dairy cow performance [33]. Meanwhile, heat stress influences the rumen microbial environment in dairy cows, leading to changes in the composition and metabolism of rumen bacteria [34]. Therefore, we hypothesized that rumen microbiota was responsible for the different lactation performance of Montbéliarde × Holstein and Holstein cows under heat stress. At the phylum level, the predominant bacterial composition of Montbéliarde × Holstein cows was similar to that of Holstein cows, with Bacteroidetes, Firmicutes, and Proteobacteria being the top three dominant phyla. Previous studies have reported significant enrichment of Proteobacteria in the rumen of cows with mastitis [35]. Similarly, we detected a higher abundance of Proteobacteria in the rumens of Holstein cows with elevated somatic cell counts. The increased relative abundance of Proteobacteria may reflect the dysbiotic ecology of the colony, which is a common feature of unhealthy populations [36]. At the family and genus levels, the relative abundances of Paraprevotellaceae, Rikenellaceae, *Allobaculum*, and *YRC22* in the rumen of Montbéliarde × Holstein cows were significantly higher than those in Holstein cows. Rikenellaceae are involved in the degradation of structural carbohydrates and starch in the rumen of dairy cows [37]. In a study on heat-tolerant dairy cows, Rikenellaceae was found to be significantly negatively correlated with rectal temperature and respiratory rate [4]. *Allobaculum* improves intestinal health and plays an important role in inflammation and metabolism [38,39]. Current research on *YRC22* (Paraprevotellaceae) is insufficient to determine its functionality. According to available reports, *YRC22* is significantly enriched in the hindgut of heat-tolerant cows and is negatively correlated with rectal temperature and respiratory rate [40]. The products of rumen microbial fermentation are VFAs, which provide most of the energy required by animals and have an important impact on ruminant production and livestock product quality. Rikenellaceae, *Allobaculum*, and *YRC22* have all been reported to be involved in VFA metabolism or positively correlated with certain VFA concentrations [4,41,42,43]. Therefore, we deduced that the rumen fermentation capacity of Montbéliarde × Holstein cows under heat stress was superior to that of Holstein cows. Furthermore, by analyzing the association between bacteria and lactation performance, we found that *YRC22* was significantly and positively correlated with milk protein content. Previous studies have established a strong correlation between the abundance of *YRC22* and the concentrations of certain amino acids in rumen fluid. Researchers have hypothesized that *YRC22* is involved, to some extent, in the production and utilization of amino acids [44]. Another study showed that *YRC22* was sensitive to different protein sources and fiber treatments, providing strong evidence of its involvement in protein processing and utilization [45]. Combined with our findings, we believe that *YRC22* has the potential to increase milk protein content.

Rumen metabolites are closely related to rumen microorganisms. We detected significantly higher levels of creatine, alpha-tocotrienol, lutein, cholesterol sulfate, phosphoenolpyruvic acid (PEP), and gentisic acid in the rumen fluid of Montbéliarde × Holstein cows than in Holstein cows. In addition to their normal physiological functions, the differential metabolites play important antioxidant and anti-inflammatory roles. As a metabolic product of amino acids (arginine, glycine, and methionine), creatine regulates cellular energy metabolism. It plays a significant role in antioxidant and anti-apoptotic responses, as well as in the scavenging of free radicals [46]. Supplementing feed with the precursor guanidinic acetic acid (GAA), which is involved in endogenous creatine synthesis, enhances ruminal antioxidant capacity and fermentation characteristics [47]. Alpha-tocotrienol, a subtype of vitamin E, exhibits a higher antioxidant activity than alpha-tocopherol. It is effective in scavenging free radicals during oxidative stress [48] and in inhibiting lipid peroxidation [49]. Lutein is a compound that is widely found in plants. According to previous studies, supplementing lactating dairy cow feed with lutein enhances the antioxidant capacity of cows, improves their lactation performance, and promotes overall health [50]. Our research indicated that the concentration of lutein in the rumen fluid of Montbéliarde × Holstein cows is significantly higher than that of Holstein cows, suggesting potentially greater absorption and utilization rates of dietary lutein by rumen microbes in Montbéliarde × Holstein cows. This phenomenon may enhance the antioxidant capacity, immunity, and overall quality of animal products obtained from Montbéliarde × Holstein cows. Cholesterol sulfate is a sulfated derivative of cholesterol that is widely found in body tissues and fluids. Cholesterol sulfate promotes cholesterol biosynthesis, influences intestinal immune responses, and limits intestinal inflammation [51,52]. In our study, a higher PEP content was detected in the rumen fluid of Montbéliarde × Holstein cows than in that of Holstein cows, which promoted glucose phosphorylation transport in rumen microorganisms and thus upregulated glycolysis. Higher PEP levels helped reduce oxidative stress, regulate energy metabolism, and improve rumen fermentation characteristics in Montbéliarde × Holstein cows under heat stress [53]. Gentisic acid possesses antioxidant and anti-inflammatory properties [54] and is usually significantly elevated in the rumen fluid of high-yielding Holstein cows [55]. In addition, significantly higher concentrations of glycocholic acid were detected in the rumen fluid of Holstein cows than in that of Montbéliarde × Holstein cows. Previous studies have shown that glycocholic acid concentration is associated with liver damage and may be an indicator of clinical ketosis in dairy cows [56]. Overall, the rumen metabolism mechanisms in response to heat stress were different in Montbéliarde × Holstein and Holstein cows, with crossbred rumen fluid containing more antioxidant and anti-inflammatory substance metabolites.

## 5. Conclusions

In conclusion, the physiological parameters and lactation performance of Montbéliarde × Holstein cows were superior to that of Holstein cows. The milk yield loss and milk somatic cell count were lower, and milk quality was higher in Montbéliarde × Holstein cows than in Holstein cows under heat stress. Meanwhile, the relative abundances of beneficial bacteria and differential metabolites with anti-inflammatory and antioxidant effects were significantly higher in Montbéliarde × Holstein cows. However, further research is essential to understand the rumen microorganisms’ interactions with their host organisms in different breeds of cows under heat stress.

## Figures and Tables

**Table 1:** Differences in physiological parameters between Montbéliarde × Holstein and Holstein cows under heat stress.

Items	H (*n* = 13)	MH (*n* = 13)	*p*-Value
Respiratory rate (bpm)	82.35 ± 2.16	71.98 ± 1.54	<0.001
Rectal temperature (°C)	39.34 ± 0.08	39.08 ± 0.05	0.011

**Table 2:** Differences in lactation performance between Montbéliarde × Holstein and Holstein cows under heat stress.

Items	H (*n* = 13)	MH (*n* = 13)	*p*-Value
Yield, kg/d			
Milk	39.55 ± 1.52	43.12 ± 1.44	0.101
Milk yield variation ^1^	−3.50 ± 1.35	−0.01 ± 0.80	0.035
4% FCM	36.46 ± 1.45	40.66 ± 1.33	0.044
Fat	1.38 ± 0.07	1.56 ± 0.06	0.048
Protein	1.25 ± 0.05	1.42 ± 0.05	0.021
Lactose	1.96 ± 0.08	2.17 ± 0.07	0.060
Milk composition, %			
Fat	3.51 ± 0.16	3.63 ± 0.08	0.493
Protein	3.17 ± 0.05	3.29 ± 0.03	0.044
Lactose	4.96 ± 0.02	5.03 ± 0.03	0.049
Total solid	12.53 ± 0.19	12.85 ± 0.10	0.145
Milk urea nitrogen, mg/dL	12.78 ± 0.40	12.34 ± 0.28	0.384
Somatic cell count, 10^4^/mL ^2^	10.33 (65.48)	4.39 (2.63)	0.002

^1^ Milk yield variation = the average milk yield under heat stress − the milk yield in one month preceding the experiment. ^2^ The data were analyzed using the Mann–Whitney U-test and presented as median (interquartile range).

**Table 3:** Alpha diversity indices of rumen bacteria between Montbéliade × Holstein and Holstein cows.

Items	H (*n* = 7)	MH (*n* = 7)	*p*-Value
Goods_coverage	0.99 (0.00)	0.99 (0.01)	0.482
Simpson	0.99 (0.01)	0.99 (0.00)	0.655
Shannon	8.91 (0.78)	8.97 (0.33)	0.406
Chao1	2074.14 (525.58)	1930.04 (270.25)	0.949

## Data Availability

The data presented in this study are available on request from the corresponding author. The data are not publicly available due to privacy.
